# Supplementary figures and images for: Elucidation of Prebiotics, Probiotics, Postbiotics, and Target from Gut Microbiota to Alleviate Obesity via Network Pharmacology Study
Source: Cells. 2022 Sep 16;11(18):2903. doi: 10.3390/cells11182903 (PMC9496669; doi:10.3390/cells11182903)

Supplementary Figure S1. The PPI networks (337 nodes and 4492 edges).

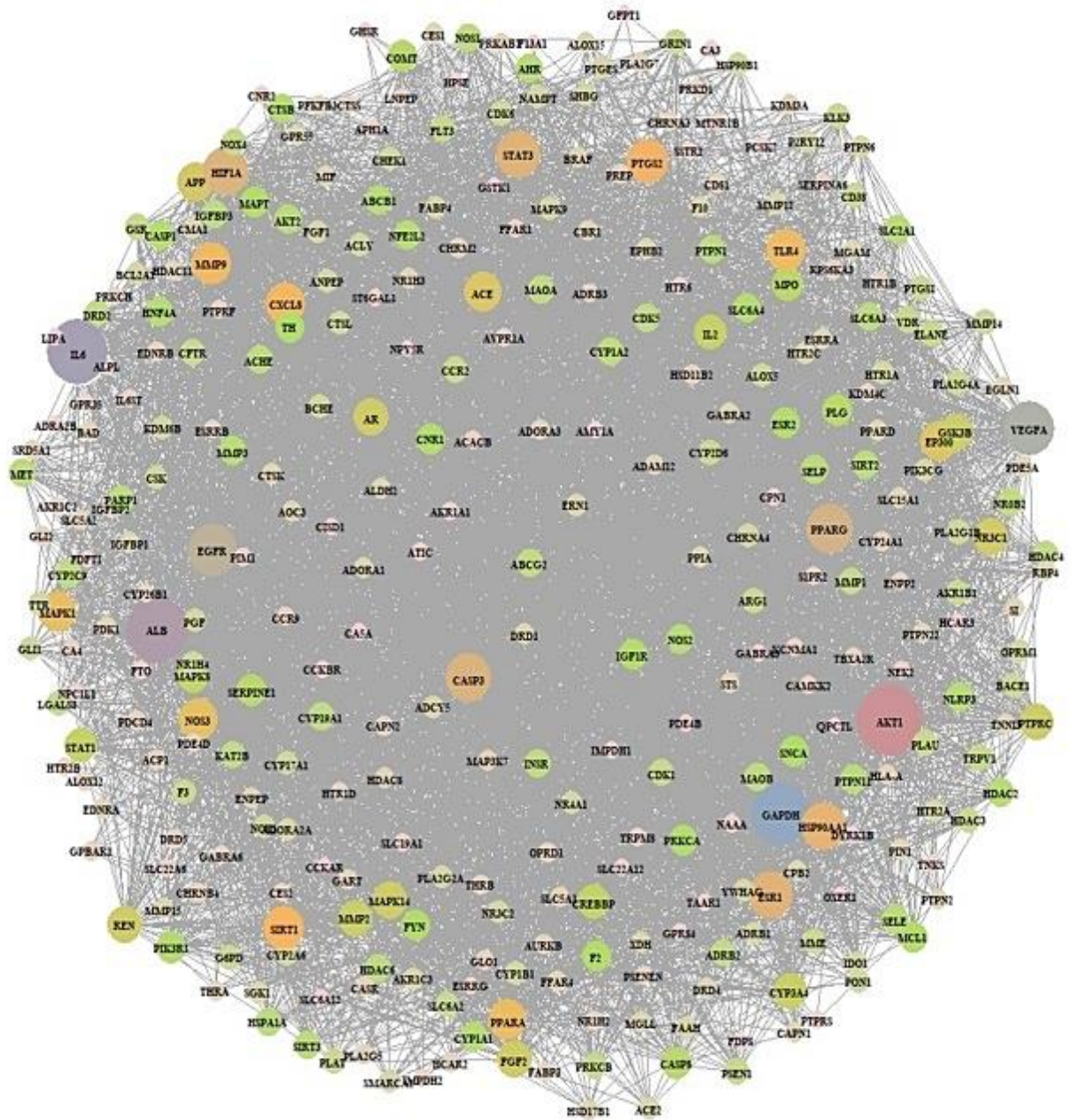

Supplement: Supplementary file 1 [file cells-11-02903-s001.zip › Supplementary Figure S1.pdf]

**Supplementary Figure S2.** The PPI networks (106 nodes and 1441 edges) of upper 30% DC value from Figure S1.

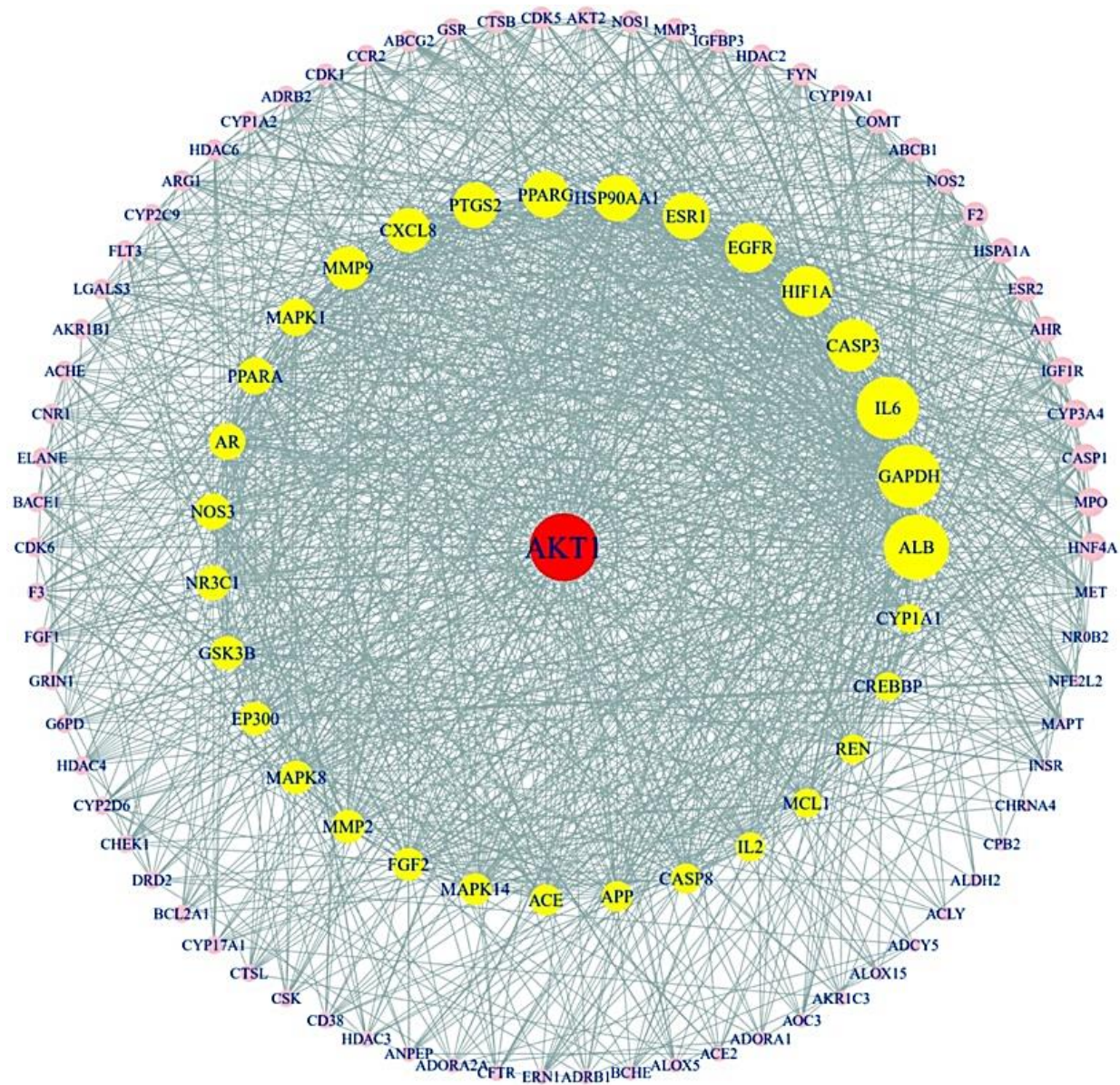

Supplement: Supplementary file 1 [file cells-11-02903-s001.zip › Supplementary Figure S2.pdf]
